# Examining Risk Factors for Mental Health During the COVID-19 Pandemic—Focusing on Older Adults in South Korea

**DOI:** 10.3389/ijph.2022.1604487

**Published:** 2022-06-24

**Authors:** Sujin Kim, Jongnam Hwang

**Affiliations:** ^1^ Korea Institute for Health and Social Affairs, Sejong, South Korea; ^2^ Division of Social Welfare and Health Administration, Wonkwang University, Iksan, South Korea

**Keywords:** Korea, mental health, older adults, COVID-19, mental health policy

## Abstract

**Objectives:** Mental health is the cornerstone of public health, particularly for older adults. There is a clear need to examine the impact of COVID-19 on mental health among older adults in South Korea, where the incidence of COVID-19 was relatively low and widespread transmission was controlled without a national lockdown.

**Methods:** This analysis included a total of 1917 participants from the Experience Survey on Healthcare Use of Older Adults, which was conducted for adults aged 65 years or older by face-to-face interview.

**Results:** The results showed that older adults with a good understanding of COVID-19 public health measures were less likely to experience mental health problems. In contrast, those with a greater risk perception of contracting COVID-19 had higher odds of experiencing tension and anxiety in addition to sadness and depression. Older adults who had a greater fear of COVID-19 and perceived higher-risk of contracting COVID-19 experienced more sleep problems.

**Conclusion:** The findings provide new evidence on the factors that influence the mental health of older adults in South Korea during the pandemic and suggest the development of policy interventions.

## Introduction

The coronavirus 2019 (COVID-19) pandemic has affected the health of older adults, leading to a public health crisis [1]. Because individuals experience fear, worry, and stress from unpredictable situations and the extensive public health measures such as social distancing, there is an increasing global concern for mental health [1]. Recent studies reported that anxiety, depression, and sleep problems increased significantly after the COVID-19 outbreak [2, 3]. Some reports suggested that older adults are one of the most vulnerable groups experiencing negative psychological and mental health consequences of the COVID-19 pandemic [2, 4–7]. The World Health Organization (WHO) warned that the spread of coronavirus would have a negative impact on mental and psychological stability especially among vulnerable groups such as older adults [6]. The United Nation (UN) also stressed that, although the COVID-19 pandemic first caused a physical health crisis, repeated blockades and quarantine measures would lead to increasingly widespread loneliness and isolation [7].

The COVID-19 pandemic could affect the mental and psychological health of older adults for various reasons. First, a fear of contracting the virus and isolating oneself out of fear could increase anxiety and depression. Older adults have a high risk of contracting COVID-19 due to decreased immunity [8]. If they are infected, they can experience a wide range of physical and mental health problems, as older adults have a higher likelihood of having a chronic disease compared to other age groups and might have a severe response to the virus [9, 10] Second, public health measures such as lockdown, social distancing, and self-isolation limit older adults’ social interactions with others, which could increase psychological problems such as anxiety and depression [5, 11]. Unlike the younger generation, which maintains their daily life through economic activities and non-face-to-face contact through various social media outlets during the pandemic, older adults have experienced social isolation and loss of emotional support [12]. Participation in social gatherings and sports clubs decreases the risk of depression of older adults as it leads to emotional and social support and exchanges with people with similar interests and social value [13]. Third, the ongoing COVID-19 pandemic has interrupted the economic flow and economic stability at the societal level and has led to unemployment and economic loss at the individual level [14], both of which can cause mental and psychological problems [15–17]. Finally, people are likely to face an overabundance of information and a rapid spread of inadequate and inaccurate information during the pandemic [18, 19]. The massive infodemic causes people to believe inaccurate information, which might increase their concerns about the virus, fear, and stress [18, 20].

Empirical studies have identified risk factors of psychological and mental health issues among the older population, including female [21], poor social relationship, no support from friends or relatives [22, 23], low socio-economic status, worsening socio-economic circumstances [24], and economic crisis [16]. A Chinese study on the mental health impacts of COVID-19 found that whereas individuals who were female and had higher education experienced greater psychological problems, a good local public health system was likely to reduce the risk of psychological problems [25]. An international study suggested that women and individuals living alone, along with pandemic-related factors such as being separated from family or close friends, were associated with a higher likelihood of depression [26]. Another study in the U.K. found that while living alone was associated with a higher likelihood of depression, financial problems, lower income, and deprivation were also associated with depression and anxiety [27]. Studies in Canada and Spain also reported that economic losses increased emotional distress among older adults [9, 28]. Considering that mental health is the cornerstone of public health, even more so among older adults, there is a clear need to address the individual and environmental factors of mental health to develop and implement appropriate policies for mental healthcare [5, 11].

Although the COVID-19 situation has rapidly changed in South Korea, the incidence of COVID-19 infection was relatively low compared with that of other countries during the first year of the global pandemic and South Korea has been considered to show successful control of the widespread transmission without a national lockdown, leading to higher adherence and trust of COVID-19-related public health measures [29, 30]. As the impacts of COVID-19 on mental health among Korean older adults are unknown, understanding which factors are associated with older adults’ mental health are valuable. mental health among older adults in South Korea is valuable. Therefore, this study aimed to examine the risk and protective factors of mental and psychological health, especially tension and anxiety, sadness and depression, and sleep problem in older adults in South Korea during the COVID-19 pandemic.

## Methods

### Data

This study used the Experience Survey on Healthcare Use of Older Adults during the COVID-19 Pandemic (the COVID-19 Survey) from the project “Korea Health Care System Performance” conducted by the Korea Institute for Health and Social Affairs (KIHASA). The purpose of this project was to assess the performance of the healthcare system at the national level and the impact of COVID-19 on health care use, social risk responses, and mental health among older adults in South Korea. The survey data was collected between 11 November 2020 and 5 December 2020 by face-to face interview using a pretested structured questionnaire and the Computer-Assisted Personal Interviews (CAPI) methodology. A total of 2000 participants were sampled in 30 regions stratified into 17 metropolitan cities and provinces to ensure national representation. As of October 2020, the number of samples was allocated in proportion to the square root according to the Ministry of Public Administration and Security’s “Resident Registration Population Status” for a total of 30 regions. The districts were randomly selected in each region, and the participants were selected based on the pre-allocation by gender and age group. The questionnaires were prepared based on the existing surveys. The questionnaires on self-reported health and chronic diseases were referenced from nationally representative survey data including the National Health and Nutrition Examination Survey and the Korea Health Panel. The questionnaire on social contact changes, fear of COVID-19, risk of COVID-19 infection, and mental health were adapted from the Survey of Health, Aging, and Retirement in Europe (SHARE). The COVID-19 survey was conducted in compliance with the COVID-19 quarantine guidelines, such as wearing a mask, and all survey participants provided written informed consent. Face-to-face data can give more accurate information compared with the surveys conducted online during the COVID-19 pandemic, which may exclude older people with limited internet access. A total of 1917 participants from the Experience Survey on Healthcare Use of Older Adults during the COVID-19 pandemic who were over the age of 65 were included in the analyses in this study. The Institutional Review Board (IRB) of the KIHASA approved the protocol (KIHASA No. 2020-76), and all participants provided written informed consent prior to the survey.

### Dependent Variables

Three aspects of mental health were assessed: tension and anxiety, sadness and depression, and sleep problems. Concerning tension and anxiety, the participants were asked, “Have you ever been nervous or anxious in the past month?” If they answered “Yes,” they were asked, “How is it compared to before the COVID-19 outbreak (January 2020)?” Respondents chose one of the three responses: “I felt less,” “I felt no different,” and “I felt more.” Similarly, questions about sadness and depression were asked. We began with “Have you ever been sad or depressed in the past month?” and then asked “How is it compared to before the COVID-19 outbreak?” Finally, concerning sleep problems, the participants were asked, “Have you slept poorly in the past month?” and if they answered “Yes,” they were asked, “How is it compared to before the COVID-19 outbreak?” This study defined an individual having a mental health problem due to the COVID-19 outbreak if they answered “I felt more” to each additional question.

### Predictor Variables

Predictor variables included possible factors affecting the mental health of older adults such as socio-demographic characteristics, health conditions, fears of COVID-19, infection with COVID-19, changes in social activities after the COVID-19 outbreak, reduction in household income, and awareness of quarantine protocols. Socio-demographic characteristics included gender, age (i.e., 65–69 years, 70–74 years, and 75 years or above), marital status (i.e., married or single/bereaved/divorced/separated), cohabitation with children, residential area (i.e., urban, or rural), economic activity status (i.e., one’s own business, employed by others for wages, or helping one’s family or relative for more than 18 h a week without receiving money), educational attainment (i.e., below and above middle school completion), and household income levels. In the overall study group, 299 participants did not respond with the amount and only answered by income category; therefore, income was divided into three groups: less than 1 million won (approximately 868 US dollars), less than 2 million won, and more than 2 million won. Health status was assessed using self-reported health (SRH) and the number of chronic diseases currently receiving treatment. SRH was divided into two categories: good (including very good, good, fair) and poor (including poor and very poor).

For the perceptions of COVID -19, three questions were asked. First, the fear of COVID-19 was assessed with the following question “How afraid are you of the Coronavirus?” and the choices were “never afraid,” “not afraid,” “moderate,” “afraid,” and “very afraid.” If the respondents answered “afraid” and “very afraid,” the answer was defined as a fear of COVID-19. Regarding the risk of COVID-19 infection, the participants were asked “How likely do you think you are going to get the coronavirus?” and “How likely do you think your family are going to get the coronavirus?” The responses were “very low,” “low,” “moderate,” “high,” and “very high.” In case the participants responded “high” or “very high” to either of the two questions, their infection risk perception was defined as high. The correlation between the individual’s own infection risk and family infection risk variable was 0.66, indicating the risk of multicollinearity; therefore, these two variables were combined (Cronbach’s alpha coefficient = 0.80). Infection with COVID-19 was measured including themselves, household members, relatives and friends, as only one participant reported that they had been infected, which is similar to the official infection rate as of 5 December 2020 (approximately 0.06%). Regarding changes in social activities, the participants were asked about how often they have done the following activities compared with before the outbreak: shopping, walking, gathering (more than 5 people), and visiting other family members such as relatives. Their response options were “less than before the outbreak,” “about similar,” and “more than before the outbreak”. The decrease in household income after the COVID-19 outbreak was defined as a decrease in the range of one-million-won unit (up to 9 million won in units of 1 million won and over 9 million won). Household income before the COVID-19 outbreak was measured retrospectively.

Awareness, trust, and satisfaction of COVID-19-related public health measures were included as predictor variables for examining the impacts on older adults’ mental health. For each of the items, the participants were asked the following questions: “I am well aware of COVID-19 related quarantine activities,” “I trust COVID-19 related quarantine activities,” and “I am satisfied with the COVID-19 related quarantine activities.” There were five possible response options: “strongly disagree,” “disagree,” “neutral,” “agree,” and “strongly agree.” Based on the respondent’ original response we re-classified the answers into two categories: agree (strongly agree, agree) and disagree (neutral, disagree, strongly disagree). The correlation between trust and satisfaction variables was found to be 0.67, indicating a multicollinearity risk. Since the regression results were similar when each variable of trust and satisfaction was included separately, the final analytic model only included trust. The analysis excluded 83 people who did not answer household income including before the COVID-19 outbreak and at the time of measurement.

### Statistical Analyses

Three different logistic regression analyses were performed on each predictor variable to identify risk factors that increased tension and anxiety, sadness and depression, and sleep problems after the COVID-19 outbreak among Korean older adults. The analyses applied the strata and weights used in sampling and were analyzed using STATA 12.0.

## Results

Table 1 presents the descriptive characteristics of the 1917 survey participants. Of the total 1917 respondents, 827 were males and 1090 were females, and the weighted percentages were 43.1% and 56.9%, respectively. Approximately 10.4% of men and 11.2% of women reported that they experienced tension and anxiety after the COVID-19 outbreak. After the COVID-19 outbreak, 11.5% of the participants stated that they experienced sadness and depression and 9.6% of the respondents stated that they had trouble sleeping. Approximately 62.5% stated that they were afraid of COVID-19 and 36.8% of the respondents reported being afraid that they or their family members were highly likely to contract COVID-19. Approximately 1.7% replied that they or their household members, relatives, or friends were infected with SARS-CoV-2. Regarding social activity, 66.6% of the total respondents stated that shopping declined after the COVID-19 outbreak. Approximately 55.8% of the total respondents stated that walking decreased after the COVID-19 outbreak. Gathering with five and more people declined by 84.4% among all participants. Approximately 81.9% of the respondents reported a decrease in family visits after the COVID-19 outbreak. Approximately 57.2% of the respondents reported that they had a good understanding of the COVID-19 public health measure, and almost 70% showed trust in the current public health measure.

**TABLE 1:** Descriptive characteristics of the study participants, (The COVID-19 survey, South Korea, 2020).

	Variables		n	%
Mental health	Tension and anxiety	Not worsened	1723	89.3
		Worsened	194	10.7
	Sadness and depression	Not worsened	1710	88.5
		Worsened	207	11.5
	Sleep problem	Not worsened	1736	90.4
		Worsened	181	9.6
Sociodemographic factors	Sex	Male	827	43.1
		Female	1090	56.9
	Age	65-69	616	32.6
		70–74	470	24.4
		75 and above	831	43.0
	Marital status	Married	1412	74.5
		Single/bereaved/divorced/separated	505	25.5
	Co-residence	With child	1532	78.7
		Without child	385	21.3
	Economic activity	Employed	671	34.9
		Economically inactive	1246	65.1
	Education	Under middle school	1044	55.4
		Middle school or higher	873	44.6
	Income	Low	971	48.6
		Middle	434	22.7
		High	512	28.6
	Residence area	Urban	1387	74.9
		Rural	530	25.1
	Self-rated health	Poor	416	21.6
		Good	1501	78.4
	Chronic condition	None	631	33.8
		1	649	34.3
		2 and more	637	31.9
COVID-19 experience	Fear of COVID-19	No	696	36.6
		Yes	1221	63.4
	Self-rated infection risk	Low	1180	62.8
		High	737	37.2
	COVID-19 infection	No	1884	98.3
		Yes	33	1.7
Social activities	Decrease in shopping	No	650	33.4
		Yes	1267	66.6
	Decrease in walking	No	840	44.2
		Yes	1077	55.8
	Decrease in gathering with 5 or more	No	310	15.6
		Yes	1607	84.4
	Decrease in visiting by other family members	No	357	18.1
		Yes	1560	81.9
Economic impact	Income decrease	No	1379	71.4
		Yes	538	28.6
Perceptions of public health measures	Understanding of public health measure	Poor	833	42.8
		Good	1084	57.2
	Trust of public health measure	Poor	554	29.4
		Good	1363	70.6

The results of the logistic regression for tension and anxiety, sadness and depression, and sleep problems are presented in Table 2. Tension and anxiety were higher among the participants who reported poor SRH (OR: 2.06, 95%CI: 1.38–3.09), were afraid of COVID-19 (OR: 2.20, 95%CI: 1.34–3.62), and thought that they or their families were at a high risk of contracting COVID-19 (OR: 2.04, 95%CI: 1.41–2.95). In addition, tension and anxiety were higher among the participants who or whose acquaintance experienced COVID-19 infection (OR: 3.14, 95%CI: 1.18–8.36). Regarding social activities, tension and anxiety was higher among participants who had decreased their amounts of shopping after the COVID-19 outbreak (OR: 2.55, 95%CI: 1.52–4.28). Tension and anxiety were lower among participants who responded that they were well aware of the COVID-19 public health measures (OR: 0.56, 95%CI: 0.39–0.79) whereas their trust in the COVID-19 public health measures did not have a statistically significant impact.

**TABLE 2:** Odds ratios (ORs) and 95% confidence intervals (CIs) from logistic regression models examining mental health after COVID-19 outbreak among older adults, (The COVID-19 survey, South Korea, 2020).

Variables		Tension and anxiety			Sadness and depression			Sleep problems		
		OR	95% CI		OR	95% CI		OR	95% CI	
Sex (ref. Male)	Female	0.98	0.68	1.42	0.68*	0.48	0.98	0.98	0.67	1.44
Age (ref. 65-69)	70–74	0.88	0.52	1.47	0.89	0.56	1.43	1.54	0.92	2.57
	75 and above	1.32	0.80	2.16	0.85	0.54	1.34	1.50	0.91	2.47
Marital status (ref. With spouse)	Without spouse	0.92	0.60	1.40	1.27	0.85	1.88	1.17	0.78	1.77
Co-residence (ref. With child)	Without child	0.76	0.44	1.32	1.03	0.61	1.74	0.78	0.44	1.40
Economic activity (ref. Employed)	Economically inactive	1.47	0.92	2.33	1.41	0.92	2.18	1.06	0.67	1.67
Education (ref. Middle school or higher)	Under middle school	0.74	0.48	1.14	0.99	0.66	1.47	0.76	0.49	1.18
Income (ref. High)	Low	0.66	0.37	1.17	0.86	0.48	1.51	0.80	0.43	1.49
	Middle	0.73	0.42	1.27	1.00	0.59	1.70	0.75	0.42	1.35
Residence (ref. Urban)	Rural	0.92	0.64	1.34	0.68*	0.47	0.99	1.19	0.82	1.73
Self-rated health (ref. Good)	Poor	2.06*	1.38	3.09	2.48*	1.72	3.59	2.53*	1.66	3.86
Chronic condition (ref. None)	1	1.17	0.76	1.81	1.67*	1.06	2.62	2.00*	1.23	3.24
	2 and more	1.14	0.70	1.85	1.42	0.87	2.33	1.56	0.92	2.65
Fear of COVID-19 (ref. No)	Yes	2.20*	1.34	3.62	1.70*	1.10	2.62	3.26*	1.92	5.52
Self-rated infection risk (ref. Low)	High	2.04*	1.41	2.95	2.38*	1.67	3.40	1.98*	1.39	2.82
COVID-19 Infection (ref. No)	Yes	3.14*	1.18	8.36	0.96	0.32	2.86	2.85*	1.02	7.95
Decrease in shopping (ref. No)	Yes	2.55*	1.52	4.28	1.31	0.86	2.01	2.06*	1.24	3.45
Decrease in walking (ref. No)	Yes	0.90	0.60	1.34	0.93	0.63	1.38	0.74	0.49	1.10
Decrease in gathering with 5 or more (ref. No)	Yes	0.67	0.36	1.22	0.88	0.45	1.71	0.72	0.37	1.41
Decrease in visiting by other family members (ref. No)	Yes	1.20	0.64	2.25	1.26	0.65	2.45	2.22*	1.07	4.59
Income decrease (ref. No)	Yes	1.30	0.90	1.89	1.48*	1.03	2.12	1.80*	1.23	2.64
Understanding of public health measure (ref. Poor)	Good	0.56*	0.39	0.79	0.56*	0.40	0.79	0.78	0.53	1.14
Trust of public health measure (ref. Poor)	Good	0.94	0.63	1.39	0.97	0.67	1.41	0.98	0.65	1.49

*p < 0.05.

Sadness and depression were lower among participants residing in rural areas, but higher among older adults with poor SRH and single chronic condition (OR: 2.48, 95%CI: 1.72–3.59; OR: 1.67, 95%CI: 1.06–2.62). Experiencing sadness and depression was higher among those who were afraid of the COVID-19 and thought they or their families were at a high risk of contracting COVID-19 (OR: 1.70, 95%CI: 1.10–2.62). The decrease of household income after the COVID-19 outbreak had a significant impact on sadness and depression (OR: 1.48, 95%CI: 1.03–2.12). Similar to the results for tension and anxiety, sadness and depression were lower among older adults who responded with a good understanding of the COVID-19 public health measures (OR: 0.56, 95%CI: 0.40–0.79); however, their trust in the COVID-19 public health measures did not have a statistically significant impact.

The frequency of sleep problems was higher among participants with poor SRH, single chronic condition, who were afraid of COVID-19, who thought that they or their families were at a high risk of contracting COVID-19, and who or whose acquaintance experienced COVID-19 infection. In addition, sleep problem was more frequent among participants who had decreased their time of shopping after the COVID-19 outbreak and had fewer visits from other family members after the COVID-19 outbreak. The odds of experiencing sleep problems were higher when household income decreased after the COVID-19 outbreak (OR: 1.80, 95%CI: 1.23–2.64). Understanding of or trust for public health measures did not have a statistically significant impact.

## Discussion

Using the recent face-to-face survey data from the Experience Survey on Healthcare Use of Older Adults during the COVID-19 pandemic, this study investigated factors affecting the mental health of Korean older adults, such as tension and anxiety, sadness and depression, and sleep problems, during the ongoing pandemic. Our results revealed that older adults who had a good understanding of COVID-19 public health measures were less likely to experience tension and anxiety. In addition, those who thought that they and their family were at a greater risk of contracting COVID-19 were more likely to experience tension and anxiety. Additionally, older adults who experienced reduced social interactions had higher odds of tension and anxiety. Older adults who had a good understanding of COVID-19 control measures were less likely to suffer from sadness and depression whereas those who perceived themselves and their family members to be at a greater risk of contracting COVID-19 had higher odds of experiencing sadness and depression. Korean older adults who had a greater fear or perceived higher-risk of contracting COVID-19 in addition to those whose household income had decreased since the outbreak were more likely to experience sleep problems. Amid the ongoing global pandemic, academic and social interests are increasing concerning the impact of COVID-19 on older adults’ mental health; however, research on the older populations in South Korea has been relatively limited. The findings from the present study provide new evidence on the factors that influence older adults’ mental health during the pandemic and suggests the implementation of policy interventions to improve their mental health.

Our study yields several important findings that can be used to inform future mental health policy for older adults. First, we found that the higher the fear of COVID-19 and the higher the perceived risk of COVID-19 for them and their family members, the greater the negative effects on their mental health, including tension and anxiety, sadness and depression, and sleep problems. These results indicate that as the epidemic continues, older adults who are more fearful and concerned about the risk of infection are continuously exposed to stressful circumstances that may further negatively affect their mental health. Previous studies also showed that quarantine policies such as containment and social distancing might affect mental health; concerns about the global pandemic and decline of social trust significantly increase loneliness and cause more serious mental health problems among older adults [31, 32]. Additionally, concerns about COVID-19 have a greater impact on individuals’ mental health than the virus itself [32].

Older adults are the most vulnerable group to COVID-19 and are at a risk of high fatality rates from the virus. Individuals aged 60–69 years and 80 years and above have fatality rates of 3.6% and 18%, respectively, which are relatively higher than rates of other age groups [9]. Therefore, negative emotions such as fear of infectious diseases, social alienation (disconnection), stigma, and stress are factors that threaten mental health of the older population, along with physical health problems caused by the COVID-19 infection [33]. Recent studies have indicated that individuals aged 60 years or above are most vulnerable to stress responses from the spread of COVID-19 [34, 35]. This adaptive response in the presence of danger eventually leads older adults to experience a wide range of mental health problems, including sadness, depression, and sleep problems. The government should develop effective strategies to provide essential information and education to improve the mental health of older adults amid the ongoing pandemic. Scientific and easy-to-understand information about infectious diseases should be provided to help reduce fear and perceived risk for ensuring the mental stability of older adults and especially older adults who experienced COVID-19 infection.

The COVID-19 pandemic is threatening the mental stability and health of older adults as daily social interactions and contacts have been restricted and contactless lifestyles are being adopted because of measures such as social distancing. As contactless lifestyles gradually became a new norm, older groups who are not proficient in using mobile phones and various digital devices began experiencing mental health problems, such as loneliness and isolation [34]. Our study found that older adults who engaged in less social activity after the outbreak, such as shopping, were more likely to experience tension and anxiety. This could be understood as the result of mental pain caused by boredom, frustration, and isolation due to the physical restrictions during quarantine. Shopping allows for interaction with others and represents a channel for stress relief for older adults [36]. Since quarantine measures such as social distancing reduce the opportunities for physical shopping, this can lead to emotional instability such as tension, nervousness, and anxiety [37]. Young people who are familiar with digital devices continue their consumption activities and fulfill their needs through online shopping, while older adults who are not familiar with digital devices might have fewer physical opportunities to buy things they need and report more experiences of social isolation compared with other age groups. Previous studies reported elevated rates of depression and loneliness among older adults during the implementation of COVID-19 restrictions; however, a lower rate of mental health issues was reported once the restrictions were eased [38, 39]. Notably, although less restrictive measures were implemented in South Korea as compared with European and North American countries, the emotional stability of older adults in South Korea was still threatened by continuous social distancing measures. To decrease social isolation and ensure emotional stability for older adults, regardless of the restrictiveness of COVID-19 control measures, developing policy interventions at both community and national levels should be considered.

Our results suggested that a sufficient understanding of quarantine activities reduced the experiences of tension, anxiety, nervousness, and depression among older adults. Studies have shown that reckless information about COVID-19 could lead to excessive concern and distrust in the healthcare system, resulting in deviations such as non-compliance with public health control rules and deterioration of physical and mental health [11, 40]. Proper understanding of public health measures can contribute toward increased mental stability by easing excessive concern and fear of COVID-19 [41]. One’s increased capacity to understand the pandemic control measures also alleviates mental distress. Therefore, promoting the effectiveness and necessity of public health measures in relation to COVID-19 could help improve the mental health of older adults [42]. An increasing number of studies confirmed that such anti-coronavirus measures such as mask and vaccine mandates have contributed to lowering the risk of COVID-19 transmission in addition to micro-close identification of clusters and contact tracing [29, 30, 43]. This may suggest that older adults who follow COVID-19-related public health measures such as full vaccination and wearing masks do not need to excessively restrict their social contact and participation, which ultimately contribute to minimizing the negative impacts of the pandemic on the mental health of older adults.

Our study also found that older adults who experienced a decrease in household income were more likely to suffer from sleep problems. This finding is consistent with previous studies, suggesting that concerns about economic losses could increase mental distress and ultimately affect and worsen sleep problems, sadness, and depression among older adults [28, 44]. Sleep is the most fundamental psychological element of health [45]. Insufficient sleep contributes not only to increase the risk of diabetes and cardiovascular diseases but also causes negative effects on health [44]. If the COVID-19 pandemic continues, it is possible that household income will continue to decrease, and therefore, various mental health problems, including sleep problems, cannot be dismissed. Financial and emotional support policies for vulnerable older adults undergoing income reduction should be considered to prevent the exacerbation of their sleep problems, as the current social protection programs for older adults are minimal [46].

Some limitations of our study should be noted. Our findings need to carefully interpret because some people might experience deterioration of mental health and some may experience improvement in any year regardless of the COVID-19 pandemic. Although the survey data was collected based on the rigorous sampling methods to ensure national representation, it is still possible that older adults with a greater fear of the virus infection did not participate in the survey interview. This could lead to unintentional underestimation of the mental health problems among Korean older adults. Due to the nature of cross-sectional data, the causality between individual’s perceptions and metal health cannot be further examined. As the COVID-19 pandemic is still far from over, collecting and monitoring information on mental health among older adults needs to be considered.

In conclusion, our study suggested that various sociodemographic factors and an individual’s perception play an important role in mental health among Korean older adults. This empirical information demonstrated the need to develop various policy interventions to provide necessary information and education with respect to COVID-19 and its related control measures and to ensure emotional stability and decrease social isolation during the COVID-19 outbreak. In addition, financial and emotional support policies need to be considered to prevent the exacerbation of sleep problems among Korean older adults.
